# Behind the flexibility: insufficient occupational injury protection of gig workers in China

**DOI:** 10.3389/fpubh.2023.1117180

**Published:** 2023-04-17

**Authors:** Xiaoxing Ran, Ying Zhao

**Affiliations:** ^1^Institute of Population Research, Peking University, Beijing, China; ^2^Medicare and Care Security Research Office, Chinese Academy of Labour and Social Security, Beijing, China

**Keywords:** gig economy, gig worker, flexibility, occupational injury, work-related injury insurance

## Abstract

**Objective:**

Occupational injury protection is essential to safeguard the basic rights of workers. This article focuses on a group of gig workers who have emerged on a large scale in recent years in China and aims to explore their status of occupational injury protection.

**Methods:**

Based on the theory of technology-institution innovation interaction, we adopted the institution analysis to assess the work-related injury protection of gig workers. The comparative study was used to evaluate three cases of occupational injury protection in China for gig workers.

**Results:**

Institutional innovation failed to respond to technological innovation and provided insufficient occupational injury protection for gig workers. The work-related injury insurance was inaccessible to gig workers due to they were not treated as employees in China. The work-related injury insurance was not available to gig workers. Although some practices were explored, shortcomings remain.

**Conclusions:**

Behind the flexibility of gig work is insufficient occupational injury protection. According to the theory of technology-institution innovation interaction, we believe the reform of work-related injury insurance is increasingly essential for improving the situation of gig workers. This research contributes to expanding understanding of gig workers' situation and may offer a reference to other countries on protecting gig workers against occupational injuries.

## 1. Introduction

In recent years, the gig economy has been a prominent topic in the field of work (1). It is subordinated to non-standard work (2), and mainly contains crowdsourced work and on-demand work (3, 4). Crowdsourced work typically refers to a range of tasks that are completed through digital platforms. These platforms are connected to a variable number of organizations and individuals around the world via the internet, with all labor requirements being posted and received, tasks being submitted and payments being made (5). On-demand work is the use of apps to communicate and perform traditional work activities such as transport, cleaning, and errands based on customers' demands (6). The apps are generally considered digital platforms, too. Hence, the gig economy includes both platforms that allow remote employment and labor transacted through platforms but provided in an appointed location (7). More work opportunities have been provided by the platform. According to a report by Didi (a travel platform in China), 1.33 million unemployed people return to the labor market to work as online drivers, with more than 12% of whom had been unemployed for more than 1 year before joining the platform. In addition, there were 1.37 million drivers from zero-employment families. In June 2020, 11.66 million registered online taxi drivers under work on the DiDi platform.

Gig workers seem to benefit a lot from the flexibility of work. However, they are facing more occupational injury risks like traffic accidents, impaired physical functioning, anxiety, depression, etc., (4, 7–9). According to the data from the project “Improving China's Institutional Capacity toward Universal Social Protection” of ILO in 2020, 2.5% of nonstandard employment workers had been involved in an accident at work, and 44.5% of these accidents occurred in the course of working on the platform. The lack of protection for gig workers is common (10–12). From January to July 2022, the number of consultations involving gig workers in Guangdong Province in China was 9,139, an increase of 50.3% year-on-year, with a monthly average of 1,305 consultations, with contract disputes, tort liability disputes, and labor disputes as the main types of consultations. In terms of tort dispute consultation, the main consultees are online car drivers and delivery workers, and the types of consultation are mostly traffic accident compensation cost standards, involving lost wages, medical (medicine) costs, repair costs, loss of transportation, etc., accounting for 30.8%, 13.6%, 13.5%, and 12.2%, respectively. It is clear that occupational injury protection has become a major concern for gig workers. With the rapid expansion of the gig economy, gig workers and their working status are in dire need of more attention.

## 2. Literature review

**Figure 1:**
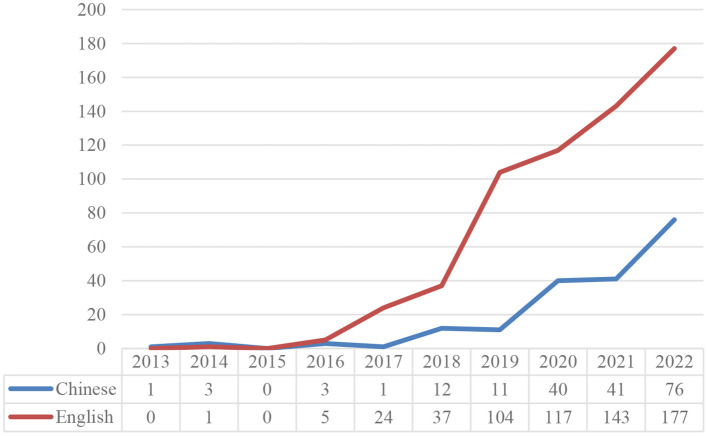
Publication of articles in Chinese and English.

In the web of science, we searched English papers with “gig work” “gig worker,” or “gig economy” as the subject words, respectively. Meanwhile, we searched the relative Chinese articles on the website of China's national knowledge infrastructure with the subject words “零工” or “零工经济,” because these two words are used more commonly in China. We only selected papers published in core Chinese journals since these papers usually have better quality. All the searches were set for the last 10 years, that is from January 2013 to December 2022. We got 608 articles in English and 188 articles in Chinese. Gig work and workers are a rising segment of the economy and have piqued the curiosity of scholars, especially in the last 3 years. From Figure 1, it is shown the number of related studies were increasing and the highest number of related articles published in 2022.

In terms of research content, existing literature focused more on gig work or workers themselves. There are three primary characteristics of gig work, including project-based compensation, temporary, and flexibility in when/how/where the work is performed (13). Among them, flexibility is not only the typical characteristic of gig work and also an important motivator for workers (7, 14). The growth in gig workers is due to the personal esteem for flexibility and freedom, favoring companies to deal with uncertainty and the advancement of digital technology (15). Because of the flexibility, gig work provides persistent ambiguity about timetables and projected compensation, as well as anxiety and overwork (16). The “anytime” and “anywhere” nature of work is gradually evolving into “always” and “everywhere” (17).

At the time level, working hours are flexible (6). Gig workers have the freedom to schedule their work time and can call on their leisure to work and monetize their time. Where there is an oversupply in the platform's virtual marketplace, platforms often direct gig workers to work for the platform by increasing the reward for their work. For example, both Uber and Lyft provide incentives to drivers in terms of the number of orders taken during a specific time, which is mostly set at peak demand or in the early hours of the morning (5). Similar regulations have prompted some workers to be willing to contribute more of their free time to the platform to increase their financial returns. In a study by Liu et al. (18), it was shown that only 8.8% of full-time online taxi drivers worked <8 h per day, and full-time online taxi drivers worked approximately 1.85 times as many hours per week as part-time online taxi drivers. In a study by Zhang Chenggang (8), 86.81% of those employed on full-time platforms worked more than 6 days per week. Extended working hours are highly likely to lead to adverse health (19). The injury rate (per 100 cumulative worker-years in a specific schedule) increased in direct proportion to the hours per day (or per week) in the workers' typical schedule, indicating a high dose-response impact (20).

At the spatial level, the flexibility leads to a tendency to bring work into their lives (21, 22). In turn leads to an increased de-boundaryization of work and living spaces, with workers accepting a gradual intensification of self-exploitation (5). The high volume of work undertaken to generate more financial income will crowd out the daily life of the workers, and the high level of work stress experienced by this group over a long time will not only affect their physical and mental health but also increase the likelihood of workplace accidents (23). For on-demand workers, single tasks are completed in relatively short periods of time and require multiple workplace changes. The risk of traffic accidents has increased. Also, gig workers are usually monitored by the platform system at all times, like the completion of each step in the work of a takeaway rider needs to be fed back to the platform system with the help of a mobile phone, and consumers can view the movement of the rider, which increases the invisible pressure (24). Working from home masks the long hours' disassociation and irregularity. The high job demand of work with less job resource always leads to burnout (25). Therefore, the flexibility of the work does not equate to improved working and living conditions (9).

In summary, the occupational injury risks faced by gig workers in a state of freedom to work stem from several sources: Firstly, the lack of clear and fixed working hours for gig workers means that the normal right to take sick leave is lost. Platforms can encourage them to work at a fast pace by setting up competitive evaluation mechanisms, reducing rest time, and granting workers no right to paid leave, which may lead to an increased incidence of illness and an increased risk of occupational injury when working with illness (26). Secondly, the lack of general workplace protectiveness, as much of the work is carried out in uncertain and private places (27). Frequent changes in workplaces also further increase the occupational injury risks faced by gig workers; Thirdly, the platform's continuous real-time assessment and evaluation of gig workers' performance are akin to a “continuous monitoring system,” which seriously affects the physical and mental health of this group when under prolonged work pressure and may easily lead to occupational injury (28). Statistics from the Shanghai Public Security Bureau Traffic Police Headquarters show that in the first half of 2017, there was an average of about one takeaway rider casualty every 2.5 days in Shanghai. In Nanjing, there were 3,242 takeaway-related traffic accidents in the first half of 2017, resulting in 2,473 injuries and three deaths. In this situation, the need for occupational injury protection for gig workers inevitably creates.

However, gig work is undertaken without the safety nets found in traditional employment (29). Gig workers are unable to count on consistent income and are barred from the labor rights afforded to employees, both of which have been worsened by the pandemic (30). Some academics believe that gig work poses challenges for social protection: one is the limited ability of gig workers to contribute to social security(including pension or other types), and the other is more companies will opt for informal employment, which in turn erodes social security models (31, 32). The gig economy relies on a workforce of independent contractors whose employment, representation, and social protection circumstances are at best uncertain, and at worst disadvantaged (14, 18, 23, 33). Due to the ambiguous status of gig workers, their protection against work-related injuries is lacking (34–36). There is still extensive debate as to whether gig workers are employees, collaborators, or a third category of workers (15–19, 33, 37–44) and this ultimately leads to the fragility of gig workers (2, 4, 7, 9, 11, 12, 23, 39, 41–43, 45–47). Several studies suggest that the social protection of gig workers is inadequate (11, 15, 31, 36). Only very few studies have separately examined the occupational injury protection of gig workers (12, 34, 36). The current work-related injury insurance has a mismatch with gig workers in China (12). Three main issues were put forward to be addressed for better occupational health research and practice: the first is if a contract is permanent or temporary; the second is whether a worker is a contractor or an employee; and the third is whether a contract involves more than one business (2). The scholar recommended that major occupational injury insurance be established within the framework of the work injury insurance system, focusing on the protection of gig workers against disability and death at work (34). Other initiatives are also put forward, for example, linking occupational injury protection to the single labor payment behavior, and the “behavior” is used as the base, rather than status (43).

It is found that existing studies examined the working status of gig workersm (4, 7, 9, 44, 45), and the analytical perspective is single which relies on the gig work and gig workers. The research on occupational injury protection for gig workers is insufficient which may lead to an incomplete description of gig workers' situation. We will explore occupational injury protection for gig workers to give a comprehensive presentation in this article, which may contribute to shedding light on the situation of gig workers.

## 3. Methods

Institution analysis and comparative study are adopted in this article. The theory of technology-institution innovation interaction sets the framework for this research, instructing us to focused on institution innovation. The institution analysis of work-related injury insurance will contribute to showing the occupational injury protection of gig workers. The comparative study is used to explore the pros and cons of three kinds of occupational injury protection and show the occupational injury protection status of gig workers furtherly.

### 3.1. Institution analysis

Based on the theory of technology-institution innovation interaction, we analyzed the institution of work-related injury insurance which is an important institutional arrangement to ensure the safety and health of employees in China. We examined the accessibility and applicability of institutions separately to see the consequences of the institution's failure to innovate. The status of occupational injury protection for gig workers is shown through institution analysis.

The theory of technology-institution innovation interaction combines technology and institution because they are closely related in the innovation systems (46, 48, 49). The adaptation of the institution to the external environment is an inevitable requirement to ensure its sustainable development. In institutional economics, technological innovation and institutional innovation are two inseparable categories that drive economic development. A rational institution is conducive to promoting technological innovation. Conversely, an institution that is inconsistent with needs may derail economic development and act as a deterrent to technological innovation (47). In Marxist economic theory, institutional factors are endogenous variables of socioeconomic development, rather than independent of it. The theory classifies technological innovation as a category of productive forces and institutional innovation as a category of relations of production, with the two having a relationship of opposition and unity. When the development of productive forces is limited by the old relations of production, innovation of institutions becomes inevitable. Technological and institutional innovation are interdependent and mutually reinforcing. In the long run, technological innovation plays an important role in promoting institutional innovation, while at the same time, institutional innovation guarantees the realization of the functions of technological innovation. The development of a country's economy and the stability of its society requires both technological and institutional innovation, thus creating a virtuous circle.

In this article, the gig economy is dependent on the input of digital technology and human capital. The flexibility of gig work is generated, but with occupational injury risks for gig workers. An effective institutional arrangement is important for protecting gig workers and then promoting the development of the gig economy. It has been shown that digital technology has given rise to the gig economy, we are wondering if is there a response to digital innovation. In a state where the technology is updated but the institution is not, what is the status of occupational injury protection for gig workers? The method of institutional analysis was adopted to give the answers in this article. We tested the applicability of the work-related injury insurance to gig workers based on the institution's condition of participation settings and protection.

### 3.2. Comparative study

In 2017, the State Council issued the “Opinions on Doing a Good Job in Employment and Entrepreneurship for the Current and Future Period,” which proposed to improve the employment and social security and other systems that adapt to the characteristics of new employment patterns; In 2020, the National Development and Reform Commission and 13 other ministries issued the “Opinions on Supporting the Healthy Development of New Business Patterns and Modes and Activating the Consumer Market to Drive Opinions on Expanding Employment,” with emphasis on exploring policies to adapt to cross-platform and multi-employer flexible employment in terms of rights and benefits protection and social security.

Data in this article are collected from the official websites of central and local governments in China, the website, and the WeChat official account platform of DiDi Travel. The documents involved are “Measures on Occupational Injury Insurance for Flexibly Employed Persons (Trial)” and “Rules for the Implementation of Occupational Injury Insurance for Flexibly Employed Persons,” “Trial Measures for County-wide Participation in Work-Related Injury Insurance for Employees in New Industrial” and Guanhuaibao. According to the information, three models of exploratory practice of occupational injury protection for gig workers were presented. The comparative study was used to make comprehensive presentations of the advantages and disadvantages of each exploratory practice. The covered subjects, financing options, and mode of operation are included to make a comparison among the three cases.

## 4. Results

The theory of technology-institution innovation interaction guides the analysis of the current state of workers' occupational injury protection. To better respond to the gig economy, the government should follow the established goals and principles for institutional innovation (50). However, work-related injury insurance failed to interact with technological innovation. Despite the particular need for workers to be covered for occupational injury protection, the current arrangement of work-related injury insurance in China does not match the situation of gig workers in many aspects, resulting in inaccessibility and a “failure” of the system. Some regions and digital companies in China have explored models of occupational injury protection for gig workers, but all have advantages and disadvantages.

### 4.1. The inaccessibility of work-related injury insurance

Work-related injury insurance is an important part of social security, providing employees with protection against work-related injuries through mutual assistance and risk-sharing. The work-related injury insurance is employer-contributed in China, and employees are not required to pay contributions. Workers can only be recognized as employees if they sign an employment contract with the employer. In terms of traditional labor relations, if the employer and the worker do not have an employment contract, very few companies will take the initiative to pay for work-related injury insurance for gig workers (48). The ambiguity of the employment status of gig workers makes it impossible to sign labor contracts with the platform companies and difficult for them to obtain insurance coverage for work-related injuries. Although platform companies have strict control over how gig workers provide services to their clients, enhancing the competitiveness of the company itself (49), by making workers recognize their status as independent contractors with flexible work, they avoid providing the range of safeguards to which workers are entitled (5). The high dependency on the platform weakens gig workers' bargaining power with the platform, and they lack a voice in the development and adjustment of platform rules that affect their interests (41).

In a state where workers' work-related injury insurance is absent, platforms can also provide safety protection for those working in the gig economy by purchasing commercial insurance, but some platform companies have failed to take effective measures. If workers are insured as individuals, this means that the cost is much higher than the group schemes offered by large employers, which is linked to economies of scale and the greater bargaining power of large employers (49). According to a survey conducted by the China Academy of Labor and Social Security Sciences, 28% of all surveyed gig workers wanted to participate in the insurance for work-related injuries, more than other insurance (51). It is no doubt that the lack of work-related injury insurance for gig workers has become an urgent problem.

### 4.2. The “failure” protection of work-related injury insurance

First, the flexibility of working time and space makes it difficult to identify work-related accidents. The work Injury Insurance Regulations in China stipulate that the injury will only be recognized as a work-related injury if it occurs during working hours, at the workplace, and is caused by work. The working hours of gig workers are often irregular, and the completion of their work may occur at any time of a day. Meanwhile, the development of digital technology allows people and establishments to complete their work even when they are spatially separated (50). This also leads to a high degree of confusion between the workplace and the living place (34). Effective screening of working hours and workplaces has become a challenge for the implementation of the work-related injury insurance. What is more, the difficulty of identifying whether a worker has been injured by work-related causes is further compounded by the irregularity of the working space and working hours, which ultimately affects the protection of the rights and interests of gig workers.

Second, it is ambiguous who is liable as an employer for the corresponding payments. The Work Injury Insurance Regulations proposed “If an employee suffers from an accidental injury at work or an occupational disease and needs to be suspended from work to receive medical treatment for the injury, during the period of suspension from work, the original salary and benefits shall remain unchanged and shall be paid monthly by the employer.” The absence of employers of gig workers is a direct result of the lack of availability of work injury benefits provided by employers.

Third, the compensation for treatment is difficult to measure when an injury occurs at work due to the unstable income of gig workers. Although the level of disability is usually used as the standard for determining compensation for treatment under work-related injury insurance, many compensation items are linked to one's monthly salary. For example, lump-sum disability benefits, disability allowances, funeral benefits, dependent's pensions, lump-sum work-related death benefit, etc., the amount of payment for these items is the worker's salary for a certain fixed number of months. As the income of gig workers is mainly based on the number of tasks completed, the income of different workers is bound to vary from one worker to another, and the monthly income of each individual is unstable, given that they are free to work at their own time and place of work. In addition, the income of gig workers is hidden, with the same worker taking on multiple jobs on multiple platforms. Hence, it is hard to determine the exact remuneration of part-time workers (12).

In short, the contents of the work-related injury insurance in terms of contributions, recognition and compensation for work-related injuries are not compatible with gig workers. Being exposed to high occupational injury risks but without the necessary protection makes gig workers more vulnerable.

### 4.3. Exploratory practice of occupational injury protection for gig workers

The lack of protection for gig workers has gradually received attention in China. Relevant government departments in various regions are gradually exploring, and some platform enterprises are also experimenting with it. Three main ways for gig workers to obtain safety protection against occupational injuries have been formed: Purchasing commercial insurance by platform, establishing separate occupational injury insurance by the government and following the existing work injury insurance system (see Table 1).

**Table 1:** Ways to achieve work injury protection for gig workers.

	**Platform companies purchase commercial insurance**	**Government establishes separate occupational injury insurance**	**Covered by the existing work-related injury insurance**
Company/region	DiDi Travel	Wujiang District, Jiangsu Province	Jiashan County, Zhejiang Province
Brands/policy documents	Guanhuaibao	“Measures on Occupational Injury Insurance for Flexibly Employed Persons (Trial)” and “Rules for the Implementation of Occupational Injury Insurance for Flexibly Employed Persons”	“Trial Measures for County-wide Participation in Work-Related Injury Insurance for Employees in New Industrial”
Covered subjects	Drivers and passengers	People work flexibly in the Wujiang area in the new economy and new business	Gig workers of new businesses operating on the platform within the administrative area of the county
Financing options	Funded by the DiDi platform	Paid by individuals, subsidized by the government	Employer contributions, no personal contributions from workers
Mode of operation	Business-led commercial insurance operating model	Government-led, commercial insurance company undertakes	Government-led social insurance model

As can be seen from Table 1, all three models are explorations of occupational injury protection for gig workers, and each has its own advantages and disadvantages. In the commercial insurance model led by platform enterprises, certain compensation can be given to online drivers in the event of accidents such as injuries from driver-rider disputes, accidental injuries and sudden death. This model is conducive to reducing the pressure of government protection, but under the conditions of different economic strength of each platform enterprise and the non-compulsory purchase of commercial insurance, it is inevitable that all gig workers will not be able to get the occupational injury protection they deserve. At the same time, commercial insurance is by nature profit-oriented, which makes it difficult for vulnerable gig workers to obtain adequate protection. Although the commercial insurance of Guanhuaibao clearly states that medical expenses, hospital meal allowance and lost wages are covered, in practice there is uncertainty as to whether the owner will receive adequate compensation. Some ambiguous treaties in the policy can lead to ambiguity in platform liability, and platforms will use their dominant position to minimize their liability and loss. The gig workers will only be able to passively accept compensation for treatment that may not be adequate, without bargaining power.

In Wujiang, Nantong and Taicang of Jiangsu Province, the government led the establishment of occupational injury insurance. This approach is more targeted, yet it also increases the economic costs and the fragmented system will pose new challenges to the development of the national social insurance system. For example, it increases the operating cost of the social insurance system, weakens the ability of the social insurance system to help each other, and increases the inequality among people. In Wujiang, for example, in terms of coverage, anyone who is flexibly employed in the region in the form of providing labor for remuneration or earnings and who is not covered by the “Regulations on work-related injury Insurance” can be insured, with no restrictions on household registration. This means that gig workers, who mobile frequently, have the opportunity to participate in the insurance and receive protection, which is important for expanding the coverage of the work-related injury insurance system. In terms of contributions and benefits, participants pay an annual lump-sum occupational injury insurance premium of RMB 180 in March each year. During the trial period, if participants are already enrolled in flexible workers' pension insurance or medical insurance, they can be subsidized by the government to the tune of RMB 120 per person each year when they are covered by the work-related injury insurance. On the whole, the premium for work-related injury insurance for flexibly employed persons is about 50% of the average local premium for work-related injury insurance, and the treatment is also at about 50% of the treatment for occupational injury insurance. In this case, although gig workers are provided with occupational injury protection, classifying gig workers as flexibly employed actually absolves platform enterprises of their responsibility to pay contributions, increasing the burden on the government and the individual workers. At the same time, as the level of compensation is lower than that of work-related injury insurance for employees, it is also a matter of concern whether gig workers can get adequate protection from it. In terms of management and operation, the government-led model with commercial insurance companies is an innovation that can not only reduce the management burden of the government, but also help to bring into play the professional advantages of commercial insurance institutions and improve the operational efficiency of the system, while it will take a longer time to test the actual operation effect.

Jiashan county of Zhejiang province tries to include gig workers directly into the target of work-related injury insurance protection. The relevant policy stipulates: e-commerce, online taxi, network food delivery, express logistics, and other new industrial enterprises operating on the platform should establish labor relations with the employees, who can participate in separate work-related injury insurance during their employment. Considering the fact that this group may have multiple jobs, each employer should separately participate in work-related injury insurance for them. The insurance is paid by the platforms, and the contribution base is calculated according to the average monthly salary of employees in the province in the previous year, and the base is adjusted according to the month following the publication of the above data by the province, and the contribution rate is tentatively set at 1.1%. Gig workers do not pay individual contributions. The taxation department has set up a “new industry injury” directory specifically for the collection of work-related injury insurance for gig workers, the collection of the directory contribution base, rate, and the amount payable by the social security agency approved and passed to the taxation authorities for collection. In this model, workers are essentially identified as employees, and platform companies are considered employers. There is no difference in coverage for gig workers and other employees. Although this initiative in Jiashan County is easy to operate and manage, it also increases the burden on platform enterprises. The work-related injury insurance should not only protect the basic rights and interests of the workers but also take into account the economic burden of the platform enterprises. At the same time, the high mobility of gig workers will also lead to a further increase in employment costs for enterprises. The direct inclusion of gig workers in the work-related injury insurance system temporarily solves the problem of participation, after which a series of issues still need to be clarified, such as whether and how to connect between work-related injury insurance platforms and different regions.

## 5. Conclusion and discussion

From the demand perspective, the flexibility of the gig economy dictates that workers have more freedom, particularly at the time and space level. However, with the incentives of platforms, gig workers are motivated to work longer hours, work harder and adapt to the uncertainty of the working space to earn more income, which leads to more occupational injury risks. The need for occupational injury protection for gig workers is a necessity. From the supply perspective, we explored the occupational injury protection of gig workers through institutional analysis and comparative studies. It is found that work-related injury insurance is inaccessible and does not apply to gig workers. Each of the protection models explored has its disadvantages. Occupational injury protection for gig workers is insufficient.

Behind the flexibility of gig work, there are increasing occupational injury risks and a lack of protection. The debate on the relationship between gig workers and platforms is the most important obstacle to gig workers' access to work-related injury insurance, and cannot be unified in the short term. However, the problem of the lack of occupational injury protection for gig workers needs to be resolved urgently. Who will provide occupational protection for gig workers? In terms of extending coverage, a government-led social insurance system is more advantageous (32). It helps to cover all gig workers in a short period of time and to achieve full coverage. The non-profit nature of social insurance allows for more adequate safety protection for gig workers. Therefore, government-led work-related injury insurance is a more reasonable option to protect gig workers from occupational injury risk. According to the theory of the interaction between technological and institutional innovation, we believe the reform of work-related injury insurance is increasingly essential to meet the needs of gig workers for occupational injury protection, and also for improving the situation of gig workers. When the system does not work as well as it should, the establishment of a new system can alleviate the problem for a while, but it is ultimately not conducive to long-term development. As the economy and society continue to develop, new issues emerge. The work-related injury insurance is not meant to be static, and in the face of changing circumstances, innovation of the institution will be the best way forward.

## Data availability statement

The original contributions presented in the study are included in the article/supplementary material, further inquiries can be directed to the corresponding author.

## Author contributions

XR conceptualized, designed the study, and performed the analysis. XR and YZ wrote the first draft and supervised the research. All authors have read and agreed to the published version of the manuscript.
